# Diabetes risk reduction diet and ovarian cancer risk: an Italian case-control study

**DOI:** 10.1007/s10552-023-01722-x

**Published:** 2023-05-24

**Authors:** Giovanna Esposito, Federica Turati, Fabio Parazzini, Livia S. A. Augustin, Diego Serraino, Eva Negri, Carlo La Vecchia

**Affiliations:** 1 Department of Clinical Sciences and Community Health, University of Milan, Via Giovanni Celoria 22, Milan, 20133 Italy; 2 Epidemiology and Biostatistics Unit, Istituto Nazionale Tumori, IRCCS Fondazione G. Pascale, Naples, Italy; 3 Unit of Cancer Epidemiology, Centro di Riferimento Oncologico (CRO), IRCCS, Aviano, Italy; 4 Department of Medical and Surgical Sciences, University of Bologna, Bologna, Italy

**Keywords:** Ovarian cancer, Case-control study, Diabetes, Diet, Glycemic index

## Abstract

**Purpose:**

To investigate the relation between a diabetes risk reduction diet (DRRD) and ovarian cancer.

**Methods:**

We used data from a multicentric case-control study conducted in Italy, including 1031 incident ovarian cancer cases and 2411 controls admitted to hospital centres for acute non-malignant disease. Subjects’ diet prior to hospital admission was collected using a validated food frequency questionnaire. Adherence to the DRRD was measured using a score based on 8 dietary components, giving higher scores for greater intakes of cereal fiber, coffee, fruit, nuts, higher polyunsaturated to saturated fatty acids ratio, lower glycemic index of diet, and lower intakes of red/processed meat, and sweetened beverages/and fruit juices. Higher scores indicated greater adherence to the DRRD. Multiple logistic regression models were fitted to calculate the odds ratios (OR) of ovarian cancer and the corresponding 95% confidence intervals (CI) for approximate quartiles of the DRRD score.

**Results:**

The DRRD score was inversely related to ovarian cancer, with an OR of 0.76 (95%CI: 0.60–0.95) for the highest versus the lowest quartile of the score (p for trend = 0.022). The exclusion of women with diabetes did not change the results (OR = 0.75, 95%CI: 0.59–0.95). Inverse associations were observed in strata of age, education, parity, menopausal status, and family history of ovarian/breast cancer.

**Conclusion:**

Higher adherence to a diet aimed at reducing the risk of diabetes was inversely associated with ovarian cancer. Further evidence from prospective investigations will be useful to support our findings.

**Supplementary Information:**

The online version contains supplementary material available at 10.1007/s10552-023-01722-x.

## Introduction

Women with diabetes mellitus are at increased risk of ovarian cancer [1], but the mechanism of such association is unclear. Hyperglycemia and hyperinsulinemia can influence ovarian cancer risk through the modulation of the insulin-like growth factor (IGF) axis [2]. Hyperinsulinemia downregulates IGF binding proteins (IGFBP) concentrations and increases free IGF-1 which promotes cellular proliferation and inhibits apoptosis [3]. In addition, inflammatory mediators and cytokines, such as interleukin-1, interleukin-6, and tumor necrosis factor, produced by the hyperglycemic state, act as growth factors by increasing angiogenesis, facilitating a tumour-favourable microenvironment, and potentially causing immune hyperactivation and tumour cell growth [4].

Diets high in glycemic index (GI) and glycemic load (GL) have been associated to type 2 diabetes risk [5, 6] and to unfavorable changes in IGFBP-3 [7]. While GI and GL were associated with an increased risk of ovarian cancer [8–10], the role of several other dietary factors on the disease is still poorly defined [11]. A dietary pattern approach allows to take into account the biologic interactions between foods and nutrients.

Rhee et al. [12] proposed a type 2 diabetes prevention diet (diabetes risk reduction diet, DRRD) characterized by high intakes of cereal fiber, polyunsaturated fatty acids, coffee, and nuts, and low intakes of carbohydrates, red and processed meat, sugar-sweetened beverages, and *trans fatty* acids. In 2020, fruit was added among the protective components and fruit juices were added among the unfavorable components of the sugar-sweetened beverages [13]. In a large cohort study from USA, the DRRD was associated with a lower risk of death from all causes, cardiovascular disease, and cancer [14]. The DRRD was also inversely associated with the risk of breast [13, 15, 16], hepatocellular [17], pancreatic [18, 19], and endometrial cancers [20]. To our knowledge, however, no study evaluated the relation between the DRRD and ovarian cancer.

In the present study, we investigated the relation between adherence to the DRRD and ovarian cancer using data from a multicentric Italian study.

## Methods

### Data Source and Study Population

Data were derived from a multicentric case-control study of ovarian cancer conducted between January 1992 and September 1999 in four Italian areas: the greater Milan area and the provinces of Pordenone, Padua, and Gorizia in northern Italy; the province of Latina in central Italy; and the urban area of Naples in southern Italy [21]. The study included 1031 cases (median age 56 years, range 18 to 79) and 2411 controls (median age 57 years, range 17 to 79).

Cases were women with a histologically confirmed incident invasive epithelial ovarian cancer admitted to the major university and general hospitals of the study areas. Controls were women admitted to the same network of hospitals as cases for a wide spectrum of acute non-malignant illnesses: traumas (26%); non-traumatic orthopedic disorders (28%); acute surgical conditions (15%); and miscellaneous other illnesses including eye, nose, ear, throat, or dental disorders (31%). Controls were excluded if they had undergone bilateral ovariectomy or if hospitalized for hormone-related, gynecological conditions, digestive tract diseases, or any clinical condition leading to long-term dietary modifications. Less than 4% of women approached refused to take part in the study and the participation rate did not vary across catchment areas or hospitals. The Ethics Committees of the Hospital “Niguarda Ca’ Granda”, Milan, and of the National Cancer Institute “Centro di Riferimento Oncologico, IRCCS”, Aviano, provided the study approval (respectively, 1125/194 and IRB-15).

Centrally trained personnel interviewed cases and controls during their hospital stay, using the same structured questionnaire and coding manual. Interviewers could not be blinded to case/control status. The questionnaire included information on sociodemographic and anthropometric features, lifestyle behaviors, personal clinical information, family (first-degree relatives) history of cancer, menstrual and reproductive factors, and use of oral contraceptives (OC) and hormone replacement therapy (HRT).

The dietary habits during the 2 years preceding cancer diagnosis (for cases) or hospitalization admission (for controls) were investigated through a valid [22] and reproducible [23, 24] food frequency questionnaire (FFQ) that included 78 items. Subjects were asked to indicate their average weekly frequency of consumption of single foods or food groups. Occasional intakes, i.e., less than once a week but at least once per month, were arbitrarily coded as 0.5 per week. To compute total energy and nutrient intake, an Italian food composition database was used [25]. Dietary GI values were derived mainly from international GI Table [26]; Italian sources were used for a few local recipes [27].

### Derivation of the DRRD score

A DRRD score was computed according to the last approach proposed in the literature [13], without trans fats, which were not included in the Italian food composition tables. The score was based on 5 dietary components favorably associated with diabetes risk, i.e., cereal fiber, coffee, fruit, ratio of polyunsaturated to saturated fatty acids, and nuts, and 3 dietary components unfavorably associated with diabetes, i.e., high dietary GI, red and processed meat, and sweetened beverages and fruit juices. We assigned scores between 1 (intake consistent with the highest diabetes risk) and 5 (intake consistent with the lowest diabetes risk) according to quintiles of consumption (derived from controls). The consumption of sugar-sweetened beverages and fruit juices was relatively uncommon in our population (61.2% of subjects reported no consumption), and thus we assigned a score of 5 to non-drinkers, a score of 3 to drinkers of ≤ 1.5 drinks per week (i.e., the median value among drinking controls), and a score of 1 to drinkers of more than 1.5 drinks per week. The consumption of nuts was reported in an open-end question of the FFQ; women declaring nut consumption were given a score of 2; otherwise, a score of 1 was assigned. Supplementary Table S1 shows the scoring of the dietary components included in the DRRD score. The individual overall DRRD score was computed as the sum of points obtained for each dietary component. Thus, the theoretical range of the final score was 8–37, with higher scores indicating greater adherence to the DRRD.

### Data analysis

We categorized the DRRD score into approximate quartiles with cutoffs derived from controls (< 22, 22–23, 24–25, and ≥ 26). Logistic regression models were fitted to calculate the odds ratios (OR) of ovarian cancer and the corresponding 95% confidence intervals (CI) for approximate quartiles of the DRRD score, using the lowest quartile as the reference category. The OR for a one-point increment in the score was also estimated. Models included terms of age (< 45, 45–54, 55–64, ≥ 65 years), center, year of interview, years of education (< 7, 7–11, ≥ 12), total energy intake (in quintiles), history of diabetes, menopausal status, parity (0–1, ≥ 2 children), use of OC, and family history of ovarian or breast cancer. The 7 women with missing menopausal status were all aged less than 49 years. Thus, they were considered as pre-menopausal.

In sensitivity analyses we repeated the main analysis excluding women with diabetes, excluding one component at a time from the overall DRRD score, and adding further adjustment for alcohol intake. We carried out stratified analyses by age, education, parity, menopausal status, and family history of ovarian/breast cancer. We tested for heterogeneity across strata using the likelihood ratio test comparing models including and not including interaction terms between the DRRD score and the stratification variable.

All statistical analyses were conducted with SAS statistical software version 9.4 (SAS Institute, Inc., Cary, NC, USA).

## Results

Table 1 shows the distribution of 1031 ovarian cancer cases and 2411 controls according to age and selected covariates. Cases had higher education and energy intake, and reported more frequently a family history of ovarian or breast cancer. The distribution of total energy intake according to the DRRD score is reported in Supplementary Table S2.

**Table 1:** Distribution of invasive epithelial ovarian cancer cases and controls according to selected covariates. Italy, 1992–1999

	Cases (N = 1031)	Controls (N = 2411)
	n (%)	n (%)
*Age *		
<45	183 (17.8)	443 (18.4)
45–54	287 (27.8)	615 (25.5)
55–64	325 (31.5)	724 (30.0)
≥65	236 (22.9)	629 (18.3)
*Education (years)*		
<7	577 (56.0)	1442 (59.8)
7–11	227 (22.0)	620 (25.7)
≥12	227 (22.0)	349 (14.5)
*Parity (children)*		
0–1	380 (36.9)	854 (35.4)
>1	651 (63.1)	1557 (64.6)
*Menopausal status*		
Pre-menopausal	346 (33.6)	803 (33.4)
Post-menopausal	683 (66.4)	1603 (66.6)
Missing	2	5
*Use of oral contraceptives*		
No	921 (89.3)	2142 (88.8)
Yes	110 (10.7)	269 (11.2)
*Family history of ovarian or breast cancer*		
No	902 (87.5)	2291 (95.0)
Yes	129 (12.5)	120 (5.0)
*History of diabetes*		
No	986 (95.6)	2324 (96.4)
Yes	45 (4.4)	87 (3.6)
*Total energy intake (quintiles)*		
Q1	142 (13.8)	547 (22.7)
Q2	188 (18.2)	499 (20.7)
Q3	227 (22.0)	462 (19.2)
Q4	242 (23.5)	446 (18.5)
Q5	232 (22.5)	457 (18.9)

Table 2 gives the OR and the corresponding 95% CI of ovarian cancer by approximate quartiles of the DRRD score, in the overall population and in non-diabetic women. High adherence to the DRRD was inversely related to ovarian cancer, with an OR for the fourth versus the first quartile of 0.76 (95% CI: 0.60–0.95; p for trend = 0.022). The corresponding OR after excluding women with diabetes was 0.75 (95% CI: 0.59–0.95). Consistent results were obtained also adding further adjustment for alcohol intake (OR = 0.75, 95% CI: 0.60–0.95).

**Table 2:** Odds ratios (OR) and corresponding 95% confidence intervals (95%CI) of invasive epithelial ovarian cancer according to the diabetes risk reduction diet (DRRD) score, in the overall population and in the subset of non-diabetic women. Italy, 1992–1999

			Overall sample	Excluding diabetic women
	Cases, N (%)	Controls, N (%)	OR (95%CI)^a^	OR (95%CI)^b^
*DRRD score, approximate quartiles*				
I (< 22)	397 (38.5)	889 (36.8)	1^c^	1^c^
II (22–23)	225 (21.8)	492 (20.4)	1.04 (0.84–1.30)	1.07 (0.85–1.34)
III (24–25)	214 (20.8)	494 (20.5)	0.94 (0.75–1.17)	0.96 (0.76–1.21)
IV (≥ 26)	195 (18.9)	536 (22.2)	0.76 (0.60–0.95)	0.75 (0.59–0.95)
χ^2^ trend (p-value)			5.25 (0.0220)	5.15 (0.0233)
One point increment in the score			0.98 (0.95-1.00)	0.97 (0.95-1.00)

^a^ Estimated from logistic regression models including terms for age, center, year of interview, education, total energy intake, history of diabetes, menopausal status, parity, use of oral contraceptives, family history of ovarian/breast cancer

^b^ Estimated from logistic regression model including same adjustment factors as reported in footnote “a” with the exception of history of diabetes

^c^ Reference category

When we excluded from the calculation each component of the score at a time, we found similar results in terms of direction and magnitude of the association; however, some of the confidence intervals became larger (Supplementary Table S3).

In subgroups analyses, no heterogeneity was found in strata of age, education, parity, menopausal status, and family history of ovarian/breast cancer (Table 3).

**Table 3:** Odds ratios (OR)^a^ and corresponding 95% confidence intervals (95%CI) of invasive epithelial ovarian cancer according to the diabetes risk reduction diet (DRRD) score in strata of selected covariates. Italy, 1992–1999

	*DRRD score, approximate quartiles*				
	I (< 22)	II (22–23)	III (24–25)	IV (≥ 26)	P for heterogeneity
Age					
<50	1.00 ^b^	0.95 (0.63–1.43)	0.75 (0.49–1.16)	0.77 (0.51–1.17)	
50–59	1.00 ^b^	1.42 (0.93–2.17)	0.99 (0.66–1.48)	0.77 (0.51–1.15)	
>59	1.00 ^b^	1.01 (0.71–1.44)	1.08 (0.76–1.54)	0.78 (0.53–1.13)	0.668
Education (years)					
<7	1.00 ^b^	1.04 (0.77–1.40)	1.02 (0.76–1.36)	0.84 (0.62–1.14)	
7–11	1.00 ^b^	0.76 (0.48–1.20)	0.85 (0.53–1.37)	0.65 (0.41–1.04)	
>11	1.00 ^b^	1.55 (0.91–2.63)	0.66 (0.38–1.14)	0.62 (0.36–1.08)	0.298
Parity (children)					
0–1	1.00 ^b^	0.95 (0.66–1.37)	0.71 (0.48–1.06)	0.62 (0.42–0.92)	
>1	1.00 ^b^	1.05 (0.80–1.40)	1.04 (0.79–1.36)	0.81 (0.61–1.07)	0.652
Menopausal status					
Pre-menopausal	1.00 ^b^	1.01 (0.69–1.49)	0.79 (0.53–1.19)	0.75 (0.51–1.11)	
Post-menopausal	1.00 ^b^	1.11 (0.84–1.45)	1.02 (0.77–1.33)	0.77 (0.58–1.03)	0.761
Family history of ovarian/breast cancer					
No	1.00 ^b^	1.08 (0.85–1.36)	0.94 (0.74–1.18)	0.77 (0.61–0.98)	
Yes	1.00 ^b^	0.72 (0.33–1.59)	0.95 (0.42–2.13)	0.79 (0.34–1.87)	0.680

^a^ Estimated from logistic regression models including terms for age, center, year of interview, education, total energy intake, history of diabetes, menopausal status, parity, use of oral contraceptives, family history of ovarian/breast cancer, unless the variable was the stratification factor

^b^ Reference category

## Discussion

In this multicentric Italian study, high adherence to the DRRD was inversely associated with ovarian cancer. After allowance for several potential confounders, including measures of endogenous estrogen exposure, use of OC, family history of ovarian/breast cancer, and total energy intake, women with the highest DRRD adherence scores had a 24% reduced risk of ovarian cancer, as compared to those with the lowest scores.

No conclusive evidence on the role of diet in ovarian cancer risk is available [11], with selected dietary factors showing limited effects on ovarian cancer occurrence [28]. According to a recent umbrella review [29], ovarian cancer risk was inversely related to black tea and calcium, and positively related to skim/low-fat milk and lactose though with a weak level of evidence.

As concerns the components of the DRRD with a protective role on diabetes, a meta-analysis including only cohort studies found no significant association between the intake of caffeine or different types of coffee and the risk of ovarian cancer [30]. Another investigation based on case-control studies documented an inverse association with decaffeinated coffee consumption [31]. A Swedish cohort study found no association between dietary phytoestrogens and the risk of ovarian cancer; however, the Swedish diet is likely to contain low amounts of phytoestrogens, from nuts, berries, beans/soy, and whole-grain bread, making it difficult to detect an association [32]. Few cohort studies reported non-significant inverse associations with fruit intake [33–36], while in other studies fruit played a favourable role against epithelial ovarian cancer [37, 38]. In a meta-analysis of 19 observational studies, fiber intake was inversely associated with ovarian cancer risk [39]. Dietary fiber may influence the disease by reducing the bioavailability of steroid hormones through changes in gut bacterial microflora, lowering availability and serum levels of estrogens, and increasing protection of lignans and other phytoestrogens [40]. In addition, dietary fiber may lower GI and GL and improve insulin sensitivity, favorably regulating IGF-1 [41]. IGF-1 stimulates cellular proliferation and inhibits apoptosis, and therefore may promote ovarian carcinogenesis [3].

Diets high in GI and GL could lead to chronic hyperinsulinemia and insulin suppresses IGFBP concentrations [7, 42]; in an Italian case-control study, ovarian cancer was inversely related to IGFBP, in particular to IGFBP-3 [43]. IGFBP-3 affect the half-life and bioavailability of IGF and may also exert IGF-1-independent effects under certain conditions [44]. At least 9 studies provided information on GI and GL and ovarian cancer risk with mixed results [8–10, 45–50]. A meta-analysis, based on 8 studies, gave a summary relative risk (RR) of 1.22 for high *versus* low GL [51]. In a previous analysis on data from the present case-control study, GI and GL were both associated with an increased ovarian cancer risk of approximately 70% [10]. Likewise, in a Canadian cohort study, high dietary GL was related to a 72% increased risk of ovarian cancer after a mean follow-up of 16 years [45]. In another study from Australia, a modest association between GL and the risk of ovarian cancer risk was found in women with overweight and/or obesity [8]. However, a cohort study from the USA found a reduced risk of ovarian cancer with higher GL [46]. Another American cohort study also observed lower risk of ovarian cancer associated with higher GI and GL [9]. The remaining 4 studies did not observe significant associations [47–50].

A meta-analysis of 8 prospective studies published up to 2011 found no appreciable association with red and processed meat [52]. Another meta-analysis published in 2011 including also case-control studies found that women with the highest intake of processed meat had a 20% increased risk of the disease and only a marginal association with red meat intake [53]. A meta-analysis of 21 observational studies indicated that consumption of total dietary fats and trans fats increased the risk of ovarian cancer [54]. A more recent meta-analysis found that also high intakes of saturated and partially monounsaturated fats, as well as cholesterol, were associated with an increased risk of ovarian cancer [55]. However, the evidence linking dietary fats with ovarian cancer risk is inconsistent across populations [56] possibly also due to the source of dietary fat consumed. Monounsaturated fatty acids from olive oil showed a modest inverse association with ovarian cancer risk in Greece, a country with the world highest consumption of olive oil [57]. High dietary fats may stimulate the secretion of estrogen [58], which can exert tumor promoting activity via mitogenic effects on ERα- positive [59, 60] or negative tumor cells [61, 62]. No associations emerged with consumption of sugar-sweetened beverages [49, 63, 64].

A pro-inflammatory diet was associated to ovarian cancer risk in studies conducted in African American [65], Australian [66], and Italian [67] women. Along this line, the original DRRD scoring system attributes higher scores to anti-inflammatory components, such as fruit and cereal fiber, and lower scores to components known to have pro-inflammatory properties, such as meat and trans fats.

Potential study limitations include the inability to account for trans fatty acids in the DRRD score since the they are not available in the Italian food composition tables. In addition, despite the window of dietary recall up to two years prior to cancer diagnosis, we could not exclude the pre-clinical symptoms modified the diet. Among the strengths, the catchment areas and the interview setting were similar for cases and controls and the participation rate was almost complete. In addition, the exclusion of women admitted for hormone-related or gynecological conditions, or any clinical condition leading to long-term modifications of diet reduced selection bias. Other strengths are the large sample size, the use of a valid [22] and reproducible [23, 24] FFQ, and the ability to control for several potential confounders.

In conclusion, this study suggests that high adherence to a diet able to reduce the risk of diabetes may also be inversely associated with ovarian cancer. Further evidence from prospective investigations is needed to support our findings.

### Electronic supplementary material

Below is the link to the electronic supplementary material.

Supplementary Material 1

## Data Availability

The dataset generated and analysed during the current study are available from the corresponding author on reasonable request.
