# Correction: Diabetes risk reduction diet and ovarian cancer risk: an Italian case–control study

**DOI:** 10.1007/s10552-024-01863-7

**Published:** 2024-02-14

**Authors:** Giovanna Esposito, Federica Turati, Fabio Parazzini, Livia S. A. Augustin, Diego Serraino, Eva Negri, Carlo La Vecchia

**Affiliations:** 1 Department of Clinical Sciences and Community Health, University of Milan, Via Giovanni Celoria 22, 20133 Milan, Italy; 2 Epidemiology and Biostatistics Unit, Istituto Nazionale Tumori, IRCCS Fondazione G. Pascale, Naples, Italy; 3 Unit of Cancer Epidemiology, Centro di Riferimento Oncologico (CRO), IRCCS, Aviano, Italy; 4 Department of Medical and Surgical Sciences, University of Bologna, Bologna, Italy

**Correction to: Cancer Causes & Control (2023) 34:769–776** 10.1007/s10552-023-01722-x

In this article the affiliation details for Livia S. A. Augustin were incorrectly given as “Department of Clinical Sciences and Community Health, University of Milan, Via Giovanni Celoria 22, Milan, 20133, Italy” but should have been “Epidemiology and Biostatistics Unit, Istituto Nazionale Tumori, IRCCS Fondazione G. Pascale, Naples, Italy”.

The original article has been corrected.

