# Supplementary material for: Diabetes risk reduction diet and ovarian cancer risk: an Italian case-control study
Source: Cancer Causes Control. 2023 May 24;34(9):769–76. doi: 10.1007/s10552-023-01722-x (PMC10363049; doi:10.1007/s10552-023-01722-x)
Supplement: Supplementary file 1 — Supplementary Material 1 [file 10552_2023_1722_MOESM1_ESM.docx]

Table S1. Scoring of the dietary components included in the diabetes risk reduction diet (DRRD) score. Italy, 1992–1999.

|  | Point obtained for each component | | | | |
| --- | --- | --- | --- | --- | --- |
|  | 1 | 2 | 3 | 4 | 5 |
|  |  |  | *Quintiles^a^* |  |  |
| Cereal fiber (g) | <2.83 | 2.83-3.65 | 3.66-4.42 | 4.43-5.51 | ≥5.52 |
| Fruit (g) | <9.75 | 9.75-14.66 | 14.67-19.25 | 19.26-26.03 | ≥26.04 |
| Coffee (n/week) | <7 | 7-13 | 14-20 | 21-27 | ≥28 |
| Polyunsaturated:saturated fats | <0.32 | 0.32-0.36 | 0.37-0.44 | 0.45-0.56 | ≥0.57 |
| Glycemic index | ≥78.27 | 75.42-78.26 | 72.73-75.41 | 69.26-72.72 | <69.26 |
| Red and processed meat (g) | ≥8.5 | 6.33-8.49 | 5.00-6.32 | 3.33-4.99 | <3.33 |
|  |  |  |  |  |  |
|  | *Other scoring systems* | | | | |
| Sweetened beverages and fruit juices (n/week) | >1.5^b^ |  | 0.5-1.5 |  | 0 |
| Nuts (proportion/week) |  | >0 |  |  |  |

^a^ Derived among controls; ^b^ Median value of consumption derived among drinking controls.

Table S2. Distribution of total energy intake according to the diabetes risk reduction diet (DRRD) score. Italy, 1992–1999.

|  | Median total energy intake (kcal/die) | |
| --- | --- | --- |
| DRRD score | Cases | Controls |
|  |  |  |
| I (<22) | 2105.1 | 1966.3 |
| II (22-23) | 2192.3 | 1966.1 |
| III (24-25) | 2223.1 | 2067.8 |
| IV (≥26) | 2197.1 | 2103.3 |

Table S3. Odds ratios (OR) and corresponding 95% confidence intervals (95%CI) of invasive epithelial ovarian cancer according to the diabetes risk reduction diet (DRRD) score after excluding each score component at a time. Italy, 1992–1999.

|  | OR^a^ (95%CI) | | | |
| --- | --- | --- | --- | --- |
| DRRD score | I (<22) | II (22-23) | III (24-25) | IV (≥26) |
| *Excluded component* |  |  |  |  |
| Cereal fiber | 1.00^b^ | 1.20 (0.96-1.49) | 0.94 (0.75-1.18) | 0.68 (0.54-0.86) |
| Fruit | 1.00^b^ | 0.97 (0.79-1.20) | 1.01 (0.81-1.26) | 0.64 (0.50-0.82) |
| Coffee | 1.00^b^ | 1.21 (0.98-1.51) | 1.07 (0.86-1.34) | 0.83 (0.66-1.05) |
| Polyunsaturated:saturated fats | 1.00^b^ | 1.11 (0.90-1.38) | 1.12 (0.90-1.40) | 0.78 (0.61-0.99) |
| Glycemic index | 1.00^b^ | 1.03 (0.83-1.28) | 1.08 (0.86-1.36) | 0.81 (0.64-1.02) |
| Red and processed meat | 1.00^b^ | 0.98 (0.78-1.23) | 1.02 (0.81.1.28) | 0.85 (0.68-1.07) |
| Sweetened beverages and fruit juices | 1.00^b^ | 0.94 (0.76-1.17) | 0.89 (0.71-1.12) | 0.68 (0.53-1.12) |
| Nuts | 1.00^b^ | 1.06 (0.85-1.32) | 0.93 (0.74-1.16) | 0.77 (0.61-0.96) |

^a^ Estimated from logistic regression model including terms for age, center, year of interview, education, total energy intake, history of diabetes, menopausal status, parity, use of oral contraceptives, family history of ovarian/breast cancer.

^b^ Reference category
